# Rural Nursing Workforce Sustainability in Australia: A Scoping Review of Global Retention Strategies

**DOI:** 10.1111/ajr.70079

**Published:** 2025-08-08

**Authors:** Atabong A. Fortabong, Kate Kloot, Natasha Reedy, Blake Peck, Daniel Terry

**Affiliations:** ^1^ School of Nursing and Midwifery University of Southern Queensland Toowoomba Queensland Australia; ^2^ School of Medicine, Faculty of Health Deakin University Warrnambool Victoria Australia; ^3^ Institute of Health and Well‐Being Federation University Australia Ballarat Victoria Australia; ^4^ Centre for Health Research University of Southern Queensland Springfield Queensland Australia

**Keywords:** health workforce, multilevel framework, organisational culture, professional development, recruitment barriers, shortages, systems

## Abstract

**Introduction:**

The global nursing shortage significantly impacts healthcare sustainability, particularly in rural Australia, where geographic isolation, professional remoteness and resource constraints amplify challenges.

**Objective:**

To map and synthesise evidence on factors influencing rural nursing workforce sustainability across individual, organisational and systemic levels.

**Design:**

A scoping review following PRISMA‐ScR guidelines was conducted. Five databases and grey literature were searched for English‐language publications (1995–2024) on rural nursing workforce sustainability. A micro‐meso‐macro conceptual framework guided the analysis of 33 publications, focusing on baccalaureate‐prepared Registered Nurses.

**Findings:**

Key determinants were identified across three levels. Micro‐level factors included personal characteristics, professional identity and safety considerations. Meso‐level factors, addressed in 32 studies, encompassed health services programmes, organisational culture, management approaches and professional development opportunities. Macro‐level factors included community engagement, educational pathways, scope of practice policies and government initiatives. Significant interconnections existed between factors across all levels.

**Discussion:**

While targeted interventions show promise, programmes need systematic evaluation using standardised frameworks. Research priorities include longitudinal effectiveness studies, economic impact analyses and the development of practical implementation tools.

**Conclusion:**

A sustainable rural nursing workforce requires integrated approaches that simultaneously address individual, organisational and systemic factors. Success depends on evidence‐based collaboration between healthcare organisations, educational institutions and government bodies to strengthen rural healthcare delivery through coordinated multilevel strategies.

**Summary**

- What is already known on this subject?
  ○ The global nursing shortage poses a significant threat to healthcare equity, particularly in rural communities where geographic isolation, professional remoteness and resource constraints amplify challenges.
  ○ Despite sustained intervention efforts, rural healthcare settings face high turnover rates and persistent nursing shortages, indicating the need for more comprehensive, theoretically informed approaches.
  ○ Previous research has typically focused on isolated intervention strategies rather than examining the complex interrelationships between individual, organisational and systemic factors affecting rural nursing workforce sustainability.
- What does this study add?
  ○ This scoping review identifies key determinants of rural nursing workforce sustainability across micro (individual), meso (organisational) and macro (systemic) levels. It reveals that nearly all studies (97%) addressed organisational factors.
  ○ The study demonstrates that effective recruitment and retention strategies must simultaneously address factors at multiple levels, as significant interactions exist between personal characteristics, organisational culture and systemic influences.
  ○ The findings highlight critical research gaps, including the need for longitudinal effectiveness studies, economic impact analyses and standardised evaluation frameworks to strengthen evidence‐based collaboration in healthcare organisations, educational institutions and government bodies.

## 1 Introduction

The global nursing workforce crisis poses a significant threat to healthcare equity, particularly in rural communities where access to essential care is compromised. Projections indicate a shortage of 7.6 million nurses by 2030, with rural areas bearing the brunt of this deficit [1, 2]. Persistent migration patterns from rural to urban areas and lower to higher‐income countries exacerbate this crisis, depleting vital healthcare resources in disadvantaged areas. As a result, rural communities face increasing challenges in maintaining adequate nursing staff, leading to significant disparities in healthcare access and delivery [3].

These rural healthcare challenges demand urgent attention in Australia, where nurses constitute 40% of the healthcare workforce [4]. With approximately seven million people, representing 28% of the nation's population, residing in rural areas [5], the projected shortfall of more than 70 000 Registered Nurses by 2035 [4, 6] poses particular risks for these communities. These workforce shortages mirror global trends and, despite sustained intervention efforts, continue to amplify health disparities between urban and rural populations.

This review defines rural healthcare settings as areas outside urban and regional centres more developed than remote areas [7]. These settings face distinct challenges shaped by geographical isolation and varying levels of infrastructure development, requiring targeted approaches to workforce sustainability.

Workforce sustainability in rural nursing encompasses the long‐term maintenance of adequate staffing levels through balanced recruitment and retention strategies that create stable, resilient healthcare teams [8]. This concept is fundamental to addressing the complex challenges faced by rural healthcare services and forms the central focus of this review.

Contemporary approaches to addressing nursing shortages reveal a persistent imbalance between recruitment and retention strategies [8]. While recruitment addresses immediate staffing requirements, achieving sustainable workforce outcomes necessitates robust retention strategies. Evidence demonstrates that targeted interventions can effectively support workforce stability, including structured mentorship programmes linking new graduates with experienced rural nurses, professional development pathways including rural‐specific specialist training, and flexible scheduling to support work‐life integration [9, 10]. However, despite these interventions, high turnover rates persist in rural healthcare settings [11], indicating the need for more comprehensive, theoretically informed approaches.

The COVID‐19 pandemic has intensified pre‐existing workforce challenges, serving as a transformative force that has exacerbated rural nursing shortages through increased workload, professional isolation and burnout. This critical juncture has highlighted the vulnerability of rural healthcare systems and underscored the need for innovative, multilevel strategies to address workforce sustainability.

The pandemic's impact on rural nursing workforce sustainability emphasises the need for a systematic theoretical framework to analyse these challenges comprehensively. A multilevel conceptual framework proposed by Terry et al. [12], supported by various researchers, including Abhicharttibutra (2023) [13], Brewer (2006) [14], Kenny (2013) [15] and Stratton (1993) [16], provides a comprehensive analytical structure for understanding these complex relationships. This framework, when modified, examines three interconnected tiers of influence: the individual nurse level (micro), which encompasses personal factors and experiences; the health service level (meso), which covers organisational structures and processes; and the broader systemic level (macro), which addresses policy and societal influences [12]. Through analysis of how factors at each level interact and affect rural nursing workforce sustainability, this structured approach enables the development of targeted, evidence‐based interventions that consider local contexts and systemic challenges [14, 15].

Scoping reviews help identify, map and categorise a topic's research extent, range and nature. This methodology is particularly suited to exploring the multifaceted challenges facing the rural nursing workforce sustainability. The review focuses on baccalaureate‐prepared Registered Nurses and those with advanced qualifications, reflecting the evolving complexity of rural healthcare delivery and the need for comprehensive solutions that address individual, organisational and systemic challenges.

### 1.1 Aim and Objectives

This scoping review seeks to answer the research question: What factors influence the sustainability of the rural nursing workforce, and how can these be effectively addressed through multilevel interventions? By systematically charting and analysing evidence, the review aims to map and synthesise factors influencing rural nursing workforce sustainability through three specific objectives: analyse individual‐level (micro) factors affecting rural nursing recruitment and retention; examine organisational‐level (meso) strategies and systems that support a sustainable rural nursing workforce; and evaluate systemic (macro) influences on rural nursing workforce sustainability.

The review aims to identify knowledge gaps and develop context‐specific recommendations for strengthening rural healthcare workforce outcomes. These recommendations will address practical implementation strategies across the three levels, particularly considering the Australian rural healthcare context while drawing insights from global experiences.

The intended outputs include an evidence map identifying key interventions at each level, a gap analysis highlighting areas requiring further research, a framework for developing integrated, multilevel approaches to workforce sustainability and actionable recommendations for policymakers, healthcare organisations and educational institutions. By adopting this comprehensive approach, the review aims to significantly address the complex challenges of rural nursing workforce sustainability in the post‐pandemic context.

## 2 Materials and Methods

This study employed a scoping review methodology as outlined by Mak and Thomas, adhering to the Preferred Reporting Items for Systematic Reviews and Meta‐Analyses extension for Scoping Reviews (PRISMA‐ScR) guidelines [17]. The investigation focused on identifying and analysing evidence‐based recruitment and retention strategies for nurses in rural healthcare settings, examining both Australian and international contexts to ensure comprehensive evidence coverage.

### 2.1 Review Design and Protocol

The researchers developed the review protocol to address the study objectives related to rural nursing workforce sustainability across three levels: individual nurse factors (micro), organisational strategies (meso) and systemic influences (macro), following the Terry et al. multilevel conceptual framework [12]. This approach guided the methodological decisions throughout the review process.

### 2.2 Search Strategy and Sources

Between February and March 2024, the researchers implemented a comprehensive search strategy across five major academic databases: Scopus, Web of Science, PubMed, APA PsycINFO and CINAHL Ultimate via EBSCOhost. Initial scoping searches confirmed the originality of this investigation by establishing the absence of existing reviews addressing rural nursing workforce sustainability using the multilevel framework approach.

The investigation examined literature from 1995 to 2024, a timeframe selected to capture established intervention approaches and emerging workforce challenges, particularly those exacerbated by recent events such as the COVID‐19 pandemic. The search methodology incorporated systematically developed key terms, including variations of ‘*nurse*’, ‘*personnel shortag*’, ‘*rural nurse*’ and ‘*strateg*’. Boolean operators combined these terms according to the search strategy detailed in the Appendix.

To enhance comprehensiveness and minimise publication bias, the researchers conducted structured searches of grey literature through Google Scholar Advanced. This approach encompassed analysis of government reports, policy documents and organisational publications across the first 22 result pages, focusing on materials relevant to the review's three‐level analytical framework.

### 2.3 Eligibility Criteria

Using the Participant, Concept, Context (PCC) framework, this review focused on baccalaureate‐prepared Registered Nurses (participants) working in rural healthcare settings (context), examining factors influencing nursing workforce sustainability (concept). The review included all nursing specialities but excluded studies of non‐baccalaureate nurses, multidisciplinary teams and metropolitan or regional practice. When providing insights into rural workforce recruitment and retention, the researchers incorporated longitudinal research tracking nursing students to professional practice. However, the researchers excluded academic theses to maintain evidence quality and manageability. This approach ensured analytical rigour while maintaining a focused examination of rural nursing workforce sustainability issues across the three levels of the research framework.

### 2.4 Study Selection Process

The research team implemented article screening and selection in two phases. The team managed eligible articles in Phase One using automated and manual approaches. The team utilised EndNote (Version 20) and the Systematic Review Accelerator (SRA) for initial duplicate removal, with subsequent manual verification to ensure thoroughness. One reviewer (AF) then screened titles and abstracts using predefined multilevel criteria. Two reviewers (AF and DT) independently conducted double screening on the JBI Sumari platform, consulting a third reviewer (NR) to resolve any disagreements through consensus.

**FIGURE 1:**
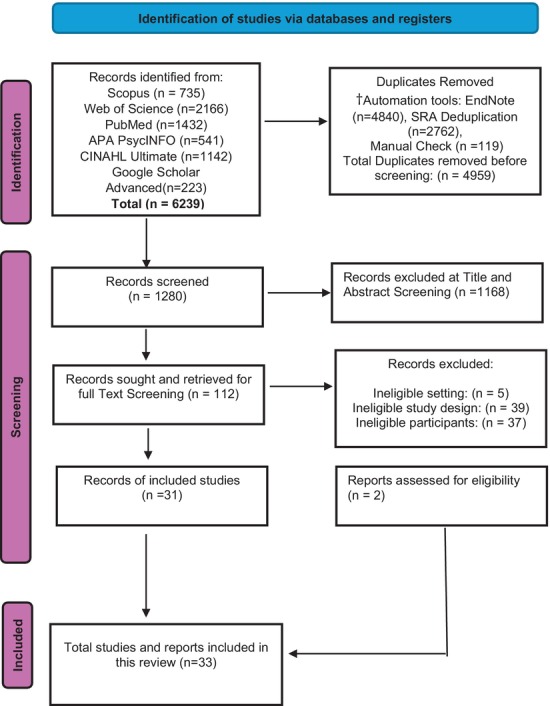
Prisma flow diagram of Search and Study Selection. ^†^To validate the automated duplicate removal process, the research team manually examined 10% of removed articles to confirm accurate duplicate identification. Source of flow chart: The PRISMA 2020 statement [18].

In Phase Two, the team performed comprehensive full‐text screening, with additional reviewers (NR and BP) available to address any screening disputes. To ensure transparency and reproducibility, the team documented all selection decisions systematically in a PRISMA flow diagram (Figure 1).

### 2.5 Data Extraction Approach

The data extraction process adhered to PRISMA‐ScR guidelines. Two reviewers (AF and DT) independently extracted data using a standardised Excel form structured according to the multilevel framework by Terry et al. [12]. This framework organised extraction across individual (micro), organisational (meso) and systemic (macro) levels. The form, pilot‐tested before implementation, captured essential study characteristics: country of origin, study design, methodology, setting and primary findings related to each framework level. Regular consultations between reviewers (NR and BP) ensured consistency and resolved discrepancies. Table 1 presents the complete framework of extracted data elements.

**TABLE 1:** Table of included studies and extracted data elements.

Study	Country	Methodology	Setting	Main results
Abhicharttibutra et al. (2023) [13, 19]	Thailand	Mixed methods (explanatory)	Rural and remote Thai community hospitals.	Generation X and Y have different reward preferences
Abhicharttibutra et al. (2023) [13, 19]	Thailand	The qualitative descriptive phase of a mixed‐method study	Rural and remote community hospitals	A combination of approaches is needed to consider different reward preferences and job satisfaction
Argent et al. (2022) [3]	Australia	Descriptive qualitative	Rural and remote Australia	A supportive team environment and effective management are retention enablers
Bao et al. (2023) [20]	China	Discrete choice experiment	Rural Guizhou Province	Equal pay, a supportive environment and contextualised incentives enhance retention
Bourque et al. (2020) [21]	Canada	Action research	Rural British Columbia	Professional networking improves the clarity and retention of the nurse practitioner role
Bragg & Bonner (2014) [22]	Australia	Grounded theory	Rural hospitals in NSW	Resignations resulted from value misalignment
Bragg & Bonner (2015) [23]	Australia	Grounded theory	Rural NSW	Organisational change must include robust resignation procedures
Brewer et al. (2006) [14]	USA	Qualitative design	Rural New York State Area	A multidimensional approach that accounts for gender and social class differentials is recommended
Crossley et al. (2023) [24]	Australia	Longitudinal qualitative	Rural Western Australia	A mismatch between rural intention and employment, skill inadequacy and family considerations are barriers
Graf et al. (2021) [25]	Australia	Mixed methods (longitudinal)	Western Australia	The graduate nurse transition program is positive, but the preceptorship model is ineffective
Hartung & Miller (2018) [26]	USA	Grounded theory (Qualitative)	Rural Pennsylvania	Improved communication methods are critical for retention
Kenny & Allenby (2013) [15]	Australia	Interpretive descriptive (Qualitative)	Rural Victoria	Long‐term, multilayered strategies needed for clinical supervision to enhance retention
Lea & Cruickshank (2017) [27]	Australia	Longitudinal case study (Qualitative)	Rural NSW and Victoria	Ineffective manager support negatively affects retention
Meyer et al. (1991) [28]	USA	Descriptive qualitative	Rural Pennsylvania	Removing financial barriers and recognising socio‐political dynamics can improve recruitment and retention
Mills et al. (2006) [9]	Australia	Project Report	Rural Australia	Mentorship exposure enhances recruitment and retention
Mills et al. (2007) [29]	Australia	Grounded theory	Rural Australia	Mentoring supports novice nurses' transition to rural practice
Mills et al. (2007) [30]	Australia	Grounded theory	Rural Australia	Live my work is complex for novice RNs. Planned mentor development opportunities may enhance retention
Molinari & Monserud (2009) [31]	USA	Quantitative (survey)	Rural USA	Nurses with rural backgrounds are more likely to stay; older nurses are less comfortable with newer immigrant groups
Opie et al. (2011) [32]	Australia	Cross‐sectional (Quantitative)	Remote health centres and hospitals	Unique stressors in different settings affect work outcomes similarly
Prengaman et al. (2017) [33]	Australia	Mixed methods	Rural Australia	NCAQ may assist in rural nurse recruitment and retention
Rohatinsky et al. (2020) [10]	Canada	Action research (Qualitative)	Rural Saskatchewan	Effective mentorship involves mentors, mentees, healthcare organisations and the community
Rose et al. (2023) [11]	Australia	Qualitative descriptive	Rural Australian hospitals	Positive RAN image and organisational support improve job satisfaction and retention
Scott & Smith (2008) [34]	USA	Action research (Qualitative)	Rural North Carolina	Group mentoring is cost‐effective and improves confidence and retention
Smith (1997)	Australia	Retrospective (Quantitative)	Large rural hospital in NSW	Multiple rotations over 12 months are ineffective for preceptors
Stewart et al. (2020) [35]	Canada	Cross‐sectional survey Quantitative	Rural and remote Canada	Personal characteristics and organisational commitments predict retention
Stratton et al. (1993) [16]	USA	Quantitative	Rural USA	Multivariate approach needed to address recruitment and retention barriers
Tamata & Mohammadnezhad (2022) [36]	Vanuatu	Qualitative	Rural Vanuatu	Poor infrastructure and equipment lead to attrition and turnover. National planning recommended
Teasley et al. (2007) [37]	USA	Action research	Rural Kentucky	Work environment influences job satisfaction and retention
Terry et al. (2024) [12]	Australia	Repeated cross‐sectional (Quantitative)	Former regional university nursing students	Micro, meso and macro factors influence rural nurse recruitment and retention
Van Haaren & Williams (2000) [38]	Australia	Opinion piece	Central Australia	CAN model resolved workforce shortages but may devalue Aboriginal health workers
Voit & Carson (2012) [39]	Australia	Descriptive qualitative	Northern Territory, Australia	Effective change management needed for post‐retirement engagement
Whiteing et al. (2022) [8]	Australia	Multiple case study	Rural and remote Victoria	Experience is perceived as more valuable than education for retention
Wright et al. (2024) [40]	Australia	Qualitative	Very remote primary health clinics	Effective WHS management is pivotal for retention

### 2.6 Analysis and Synthesis Method

The reviewers employed a structured approach to examine framework‐level patterns and relationships, consistent with Pollock et al. best practice recommendations for scoping reviews [41]. The reviewers applied deductive content analysis to identify key themes within each level of the multilevel framework (micro, meso, macro) while maintaining precise alignment with the study objectives. Table 2 provides a summary of the included studies and their key characteristics.

**TABLE 2:** Key characteristics of included studies.

Study	Concept	Micro‐level factors (*n* = 19)	Meso‐level factors (*n* = 32)	Macro‐level factors (*n* = 11)
Abhicharttibutra et al. (2023) [13, 19]	Retention and Turnover	✓	✓	─
Abhicharttibutra et al. (2023) [13, 19]	Retention	✓	✓	✓
Argent et al. (2022) [3]	Retention and Turnover	─	✓	─
Bao et al. (2023) [20]	Retention and Turnover	✓	✓	✓
Bourque et al. (2020)	Recruitment and Retention	─	✓	─
Bragg & Bonner (2014) [22]	Retention	─	✓	─
Bragg & Bonner (2015) [23]	Turnover	─	✓	─
Brewer et al. (2006) [14]	Recruitment and Retention	✓	✓	✓
Crossley et al. (2023) [24]	Recruitment and Retention	✓	✓	✓
Graf et al. (2021) [25]	Retention	✓	✓	─
Hartung & Miller (2018) [26]	Retention	─	✓	─
Kenny A, Allenby A. 2013 [15]	Retention	─	✓	─
Lea & Cruickshank (2017) [27]	Retention and Turnover	─	✓	─
Meyer et al. (1991) [28]	Recruitment	✓	✓	✓
Mills et al. (2006) [9]	Recruitment and Retention	✓	✓	─
Mills et al. (2007) [29, 30]	Retention	✓	✓	─
Mills et al. (2007) [29, 30]	Retention	✓	✓	─
Molinari & Monserud (2009) [31]	Retention	✓	✓	─
Opie et al. (2011) [32]	Turnover	─	✓	─
Prengaman et al. (2017) [33]	Recruitment and Retention	✓	✓	─
Rohatinsky et al. (2020) [10]	Retention	✓	✓	─
Rose et al. (2023) [11]	Recruitment and Retention	✓	✓	✓
Scott & Smith (2008) [34]	Retention	─	✓	─
Smith (1997)	Retention	─	✓	─
Stewart et al. (2020) [35]	Retention	✓	✓	─
Stratton et al. (1993) [16]	Recruitment and Retention	─	✓	✓
Tamata & Mohammadnezhad (2022) [36]	Recruitment, Retention and Turnover	✓	✓	✓
Teasley et al. (2007) [37]	Retention and Turnover	─	✓	─
Terry et al. (2024) [12]	Retention and Turnover	✓	✓	─
Van Haaren & Williams (2000) [38]	Retention	─	✓	✓
Voit & Carson (2012) [39]	Retention	─	✓	─
Whiteing et al. (2022) [8]	Retention and Turnover	✓	✓	✓
Wright et al. (2024) [40]	Turnover and Retention	✓	✓	✓

The analysis process involved the initial coding of findings according to the three framework levels, identifying recurring themes within each level, examining relationships between factors across different levels and integrating quantitative findings (using descriptive statistics) and qualitative results (using narrative synthesis). Regular team reviews validated thematic categorisation and confirmed hypothesis alignment [17]. This approach enabled systematic examination of rural nursing workforce factors while maintaining analytical rigour through independent review, structured analysis and team validation.

### 2.7 Quality Assessment

While formal quality assessment is not mandatory for scoping reviews, the reviewers documented the methodological characteristics of the included studies to provide context for interpretation. This documentation included study design, sample size, methodology noted by authors and relevance to the multilevel framework.

## 3 Results

### 3.1 Overview of Included Studies

This scoping review synthesised evidence from 33 studies examining rural nursing workforce sustainability. The analysis incorporated diverse methodological perspectives, including 18 qualitative studies (54.5%), nine quantitative studies (27.3%), four mixed‐methods studies (12.1%), one project report (3.0%) and one opinion piece (3.0%). These studies span multiple geographical contexts, with notable contributions from Australia (57.6%), the United States (21.2%), Canada (9.1%) and other international settings (12.1%).

### 3.2 Framework Application

**FIGURE 2:**
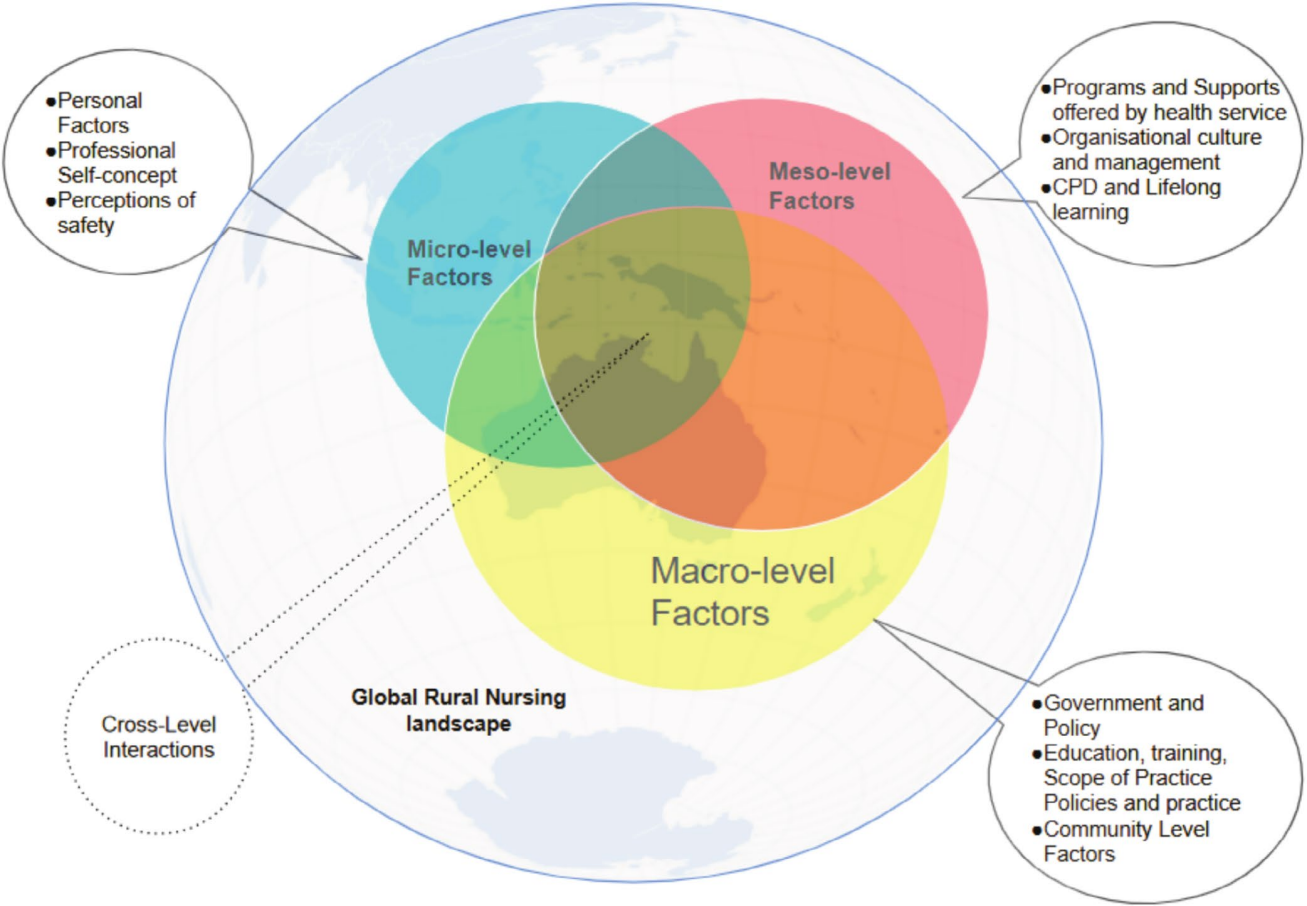
Conceptualisation of micro, meso and macro level factors interactions.

The analysis identified distinct yet interrelated determinants across the framework's three levels. Of the 33 studies reviewed, 19 (57.6%) examined micro‐level influences, 32 (97.0%) investigated meso‐level factors and 11 (33.3%) addressed macro‐level considerations. The prominence of meso‐level factors, addressed in nearly all studies, emphasises their central role in sustaining the rural nursing workforce. Figure 2 illustrates the distribution and relationships of these factors within the conceptual framework.

### 3.3 Micro‐Level Factors

The analysis identified three key micro‐level factors influencing rural nursing workforce sustainability: personal characteristics, professional self‐concept and safety perceptions.

#### 3.3.1 Personal Characteristics

Personal characteristics significantly shape workforce sustainability. Research demonstrates that intrinsic motivation, age demographics, reward preferences, family circumstances, cultural background and marital status influence nurses' decisions to maintain rural positions [13, 14, 28, 35, 40]. While senior nursing staff demonstrate higher turnover rates [14, 19], nurses with rural backgrounds consistently show improved retention rates [31]. Multiple studies posit that lifestyle preferences, spousal satisfaction and community accessibility are crucial determinants for long‐term retention [33].

#### 3.3.2 Professional Self‐Concept

Professional self‐concept substantially impacts workforce stability in rural healthcare settings. Evidence consistently indicates that nurses with higher confidence in their professional capabilities maintain more significant retention rates in rural settings [12, 28, 40]. However, multiple studies highlight that early‐career nurses frequently report feeling underprepared for the comprehensive scope of rural nursing practice [8, 11, 24, 25]. Research findings emphasise that practical experience, rather than formal education alone, better facilitates successful transitions to rural nursing practice [8].

#### 3.3.3 Safety Perceptions

Safety perceptions within healthcare facilities and communities represent the third critical determinant of workforce sustainability. Studies provide moderate evidence that implementing established safety protocols, appropriate equipment availability and effective community integration demonstrates enhanced retention outcomes [20, 30, 36]. Furthermore, comprehensive support systems, including mental health resources, mentorship programmes and peer networks, positively influence safety perceptions and contribute to sustained workforce stability [9, 10, 11, 29].

These three micro‐level factors form an interconnected framework that shapes rural nursing workforce outcomes. Understanding these relationships enables the development of targeted strategies for sustainable rural healthcare delivery, particularly when integrated with meso‐ and macro‐level interventions.

### 3.4 Meso‐Level Factors

The analysis identified three primary meso‐level factors influencing rural nursing workforce sustainability: health service programmes and support, organisational culture and management approaches and professional development opportunities.

#### 3.4.1 Health Service Programs and Support

Health service programs and support significantly shape rural nursing workforce outcomes. Strong evidence from multiple studies indicates that organisations implementing comprehensive workforce strategies demonstrate enhanced sustainability [14, 19, 29, 39], primarily through pre‐ and post‐retirement engagement initiatives, competitive remuneration packages and structured mentorship programs. Targeted approaches to address accommodation and family needs have proven consistently effective [10, 11, 20], with the Nursing Community Apgar Questionnaire (NCAQ) [33] providing valuable assessment capabilities for recruitment and retention efforts.

#### 3.4.2 Organisational Culture and Management Approaches

Organisational culture and management approaches emerge as equally crucial determinants of workforce stability. Substantial evidence indicates that supportive work environments and stable, flexible employment arrangements substantially influence workforce retention [3, 8, 32, 37]. Structured transition programmes emerged as particularly valuable in supporting early‐career nurses navigating rural healthcare settings, with consistent findings across multiple studies [24, 25, 27, 42].

#### 3.4.3 Professional Development Opportunities

Continuous Professional Development (CPD) and lifelong learning opportunities constitute the third critical factor affecting workforce sustainability. Rural nurses working in organisations with robust CPD programs consistently report higher job satisfaction and decreased professional isolation and attrition [8, 13, 40]. However, implementing comprehensive CPD programs in rural healthcare settings presents ongoing challenges due to resource constraints and staffing limitations [11, 24], highlighting an area where evidence for successful implementation strategies remains limited.

These meso‐level factors demonstrate significant interdependence, indicating that effective recruitment and retention strategies must adopt an integrated approach addressing multiple organisational dimensions. This multifaceted perspective provides essential insights for healthcare organisations seeking to strengthen their rural nursing workforce sustainability, particularly when considering the unique challenges and opportunities in rural healthcare environments.

### 3.5 Macro‐Level Factors

The analysis identified three critical macro‐level factors influencing rural nursing workforce sustainability: community engagement, educational pathways, and scope of practice policies and government initiatives. These systemic elements collectively shape the landscape of rural nursing practice and workforce development.

#### 3.5.1 Community Engagement

Community engagement emerges as a foundational macro‐level factor shaping rural nursing workforce sustainability. Multiple studies provide moderate evidence that establishing nursing education programmes within rural communities and early exposure to rural healthcare settings significantly influence nurses' career trajectories towards rural practice [14, 28]. These community‐based approaches create sustainable pathways that contribute to long‐term workforce development and stability, fostering a culture of rural healthcare excellence.

#### 3.5.2 Educational Pathways and Scope of Practice Policies

Educational pathways and scope of practice policies constitute interconnected macro‐level influences on rural nursing workforce development. Rural‐focused and targeted educational pathways demonstrate the potential for enhancing retention through structured professional advancement opportunities [16, 36, 38]. Furthermore, policies that support advanced practice roles and continuous professional development positively correlate with job satisfaction and long‐term retention [8, 11]. Implementing these educational frameworks, particularly in rural contexts, shows promising results for enhanced rural nursing workforce sustainability and professional growth.

#### 3.5.3 Government Policy Initiatives

These initiatives are pivotal in addressing systemic challenges in rural healthcare delivery. Strategic approaches with demonstrated positive outcomes include adjusting nursing school enrolment quotas, reducing financial barriers to nursing education and implementing targeted rural allocation programmes for new graduates [16, 24, 28, 36]. Additionally, infrastructure investments and financial incentives have the potential to enhance the appeal of rural positions through improved working conditions and career development opportunities [20, 36, 40]. These economic policies significantly influence regional employment markets and location decisions [16], while targeted funding directly impacts working conditions and compensation structures [8, 19]. Notably, initiatives focused on developing local workforce capacity and reducing barriers to nursing education demonstrate promising outcomes for sustained recruitment and retention.

### 3.6 Cross‐Level Interactions

The analysis revealed significant interactions between factors operating across different framework levels. For example, personal characteristics (micro‐level) interact with organisational culture (meso‐level) to influence workforce outcomes, as evidenced by studies showing how individual motivations interact with organisational values to enhance or impede retention [12, 22, 23, 28]. Similarly, educational pathways (macro‐level) influence professional self‐concept (micro‐level) through targeted rural nursing curricula that better prepare nurses for rural practice demands [8, 24].

Several studies demonstrated how health service programmes (meso‐level) could be optimised when aligned with government initiatives (macro‐level), particularly in areas of financial incentives and professional development opportunities [16, 36, 40]. The most effective workforce sustainability strategies identified in the literature address factors across multiple levels simultaneously, recognising the interconnected nature of these influences.

### 3.7 Evidence Gaps

The analysis identified several areas where evidence remains limited or inconsistent. First, longitudinal studies examining the long‐term effectiveness of specific interventions are notably scarce, with most research providing cross‐sectional analyses. Second, economic evaluations comparing the cost‐effectiveness of different recruitment and retention strategies are primarily absent, limiting evidence‐based resource allocation decisions. Third, research explicitly examining the interaction effects between micro, meso and macro factors is underdeveloped, with most studies focusing primarily on one level. Finally, the impact of technological advances on rural nursing practice and workforce sustainability represents an emerging area requiring further investigation, particularly given the evolution of post‐pandemic healthcare.

## 4 Discussion

### 4.1 Summary of Key Findings

This scoping review reveals the complex interrelationships between factors affecting rural nursing workforce sustainability across micro, meso and macro levels, demonstrating the limitations of single‐level analyses. Using the Terry et al. multilevel framework [12], this review's analysis illuminates the dynamic interactions between individual characteristics, organisational structures and systemic influences that collectively shape rural nursing recruitment, practice and retention. These findings offer significant implications for evidence‐based policy development and practice improvement.

### 4.2 Interpretation Within Multilevel Framework

#### 4.2.1 Micro‐Level Factors and Their Implications

Rural nurses face distinct challenges regarding personal factors, professional self‐concept and safety perceptions [30]. Their expanded scope of practice and resource constraints interact directly with organisational structures and policy frameworks, creating unique professional demands. Personal characteristics, including rural background, family circumstances and professional confidence, consistently influence retention outcomes across diverse contexts. These findings suggest that recruitment strategies targeting candidates with rural connections may yield more substantial retention outcomes, while professional development initiatives should focus on building confidence in rural practice capabilities.

#### 4.2.2 Meso‐Level Considerations and Organisational Implications

This review's meso‐level analysis, supported by 32 of the 33 reviewed publications, demonstrates how organisational culture and management practices are critical mediators linking individual experiences with systemic influences. While organisational support systems remain essential across developed and developing nations, their effectiveness depends on alignment with policy frameworks and individual needs. Professional isolation and resource limitations [43, 44] illustrate how structural issues affect individual experiences and career decisions. Successful organisational interventions, particularly mentorship and leadership programmes [3, 8, 27], effectively bridge policy objectives with professional development needs, suggesting that organisations should prioritise these approaches in rural settings.

#### 4.2.3 Macro‐Level Influences and Policy Implications

Macro‐level factors, including community integration, educational pathways and government policies, establish the essential context within which organisational practices and individual career decisions operate. This finding aligns with the Terry et al. framework [12], demonstrating how systemic factors influence individual outcomes through organisational structures. Rural workforce maldistribution [35] exemplifies how macro‐level policies shape organisational capabilities and professional career trajectories. These patterns appear consistent across high‐income countries, including Canada, the United Kingdom and New Zealand, indicating universal relationships between organisational structures and systemic policy frameworks.

#### 4.2.4 Cross‐Level Interactions

The review findings emphasise that these factors operate as interconnected systems requiring interventions that simultaneously address multiple levels. While macro‐level educational policies create opportunities, their success fundamentally depends on organisational implementation and alignment with individual career aspirations. Similarly, retention strategies must consider policy constraints, organisational capabilities and professional development needs to achieve sustainable outcomes. This interconnectedness explains why isolated interventions often demonstrate limited effectiveness, while comprehensive approaches addressing multiple levels will likely show more substantial outcomes.

### 4.3 Integration With Existing Literature

This review's findings align with broader healthcare workforce literature, highlighting nursing‐specific considerations in rural contexts. The challenges mirror those facing other rural health professionals, including physicians [45] and allied health practitioners [46], particularly regarding professional isolation, scope of practice concerns and continuing education access. However, nursing‐specific factors emerge, including the critical importance of professional self‐concept and safety perceptions within expanded practice roles.

The review findings demonstrate alignment with WHO global health workforce sustainability frameworks [2], emphasising coordinated approaches to recruitment, education, deployment and retention. However, this review's analysis extends these frameworks by identifying how policy initiatives influence organisational capacity and individual decision‐making in rural nursing contexts. The intensification of workforce challenges during the COVID‐19 pandemic further underscores the vulnerability of rural healthcare systems and the need for resilient, multilevel workforce strategies.

### 4.4 Evidence‐Based Programme Evaluation

Research demonstrates that effective rural nurse retention requires an integrated, stakeholder‐driven approach [47]. Programme evaluations offer key insights into successful retention strategies and implementation considerations. The Whole‐of‐Person Retention Improvement Framework (WoP‐RIF) in rural Victoria exemplifies an evidence‐based intervention addressing multiple framework levels simultaneously. This comprehensive framework combines professional development initiatives with support for personal well‐being. Evaluations show improved staff satisfaction, engagement and retention rates [48, 49], confirming that successful retention strategies must address both professional and personal aspects of rural nursing practice.

The Australian Rural Health Multidisciplinary Training (RHMT) programme demonstrates effective workforce development through educational pathways. The programme aims to improve rural health professional recruitment and retention through sustained rural clinical placements. Evaluation evidence shows that extended rural exposure during training significantly influences long‐term workforce outcomes. These findings align with international reviews identifying financial incentives, professional advancement opportunities and workplace support systems as key elements for workforce stability [50].

Evidence from these established programmes enables healthcare organisations and policymakers to implement data‐driven interventions [47]. Emerging evaluation frameworks measuring multiple dimensions, including workforce retention rates [44], financial indicators such as turnover costs, and quality indicators related to professional satisfaction, provide robust assessment tools for programme effectiveness. This growing knowledge base supports the development of contextually adapted interventions for diverse rural healthcare settings.

### 4.5 Policy Implications and Implementation Considerations

#### 4.5.1 Evidence‐Informed Policy Development

Contemporary evidence demonstrates that effective rural healthcare workforce planning requires a structured, evidence‐based approach grounded in multilevel analysis [47]. Systematic evaluation of retention interventions should directly inform policy development [44], while successful implementation demands thoughtful adaptation to local healthcare environments [45]. The rural pipeline concept exemplifies these evidence‐based principles by strengthening recruitment and retention throughout healthcare careers. This integrated approach aligns with WHO (2021) guidelines on health workforce development, emphasising coordinated policy responses and sustained investment in immediate needs and long‐term workforce sustainability [2].

#### 4.5.2 Implementation Strategies

Sustainable rural healthcare delivery requires careful alignment between service models and workforce retention strategies. While nurse‐led models effectively address rural healthcare access barriers, workforce turnover challenges their sustainability [46]. Research consistently shows that effective policy development requires a nuanced understanding of attraction and retention factors [50, 51, 52]. Therefore, policy success depends on optimising service delivery models and workforce sustainability mechanisms.

Implementation requires particular attention to local context, available resources and meaningful stakeholder engagement. Healthcare organisations need adaptive strategies that respond to emerging evidence while focusing on long‐term sustainability goals. Successful adaptation requires:

- Continuous evaluation of intervention effectiveness
- Regular assessment of workforce needs and preferences
- Strategic alignment of resources with organisational objectives
- Integration of retention initiatives within broader service planning

Prioritisation should focus on high‐impact interventions addressing multiple framework levels simultaneously, particularly those demonstrating cost‐effectiveness and contextual adaptability.

### 4.6 Contextualising Within the Australian Rural Nursing Landscape

This review offers a comprehensive examination of the international literature, providing insights relevant to Australia's unique rural nursing environment, which is shaped by its distinctive geography, demographics and policy frameworks. Rural areas in Australia present specific logistical and professional challenges that amplify the micro‐, meso‐ and macro‐level factors discussed. For instance, the broader scope of practice expected of rural nurses emphasises the need for tailored professional development [8, 40, 46].

Recruitment strategies that favour candidates from rural backgrounds and promote community integration align with the ‘rural pipeline’ approach in Australia, exemplified by initiatives such as the RHMT program. The program's success in facilitating sustained clinical placements and enhancing long‐term workforce retention demonstrates the effectiveness of macro‐level policy interventions [24, 25]. At the meso level, the Wop‐RIF illustrates organisational strategies tailored to meet both the professional and personal needs of rural nurses [48, 49]. These examples confirm the value of the multilevel framework and emphasise the importance of context‐sensitive policy implementation, especially within the health system and its regional workforce disparities observed across Australia [43, 44].

Additionally, it is vital to recognise the crucial role of the indigenous nursing workforce. Aboriginal and Torres Strait Islander peoples remain significantly underrepresented in nursing, despite their communities facing disproportionate health challenges in rural and remote areas [4, 38]. Recruitment and retention strategies must therefore include culturally safe pathways that support Indigenous students and professionals. The indigenous nursing pipeline, which encompasses outreach, education, mentorship and employment, requires ongoing investment to address systemic barriers and promote equity [43, 53]. The greater integration of Indigenous perspectives into nursing curricula and the development of community‐based placements can strengthen the links between Indigenous nurses and their communities, thereby enhancing workforce sustainability and improving health outcomes [53]. These initiatives align with macro‐level policy and meso‐level organisational strategies that need adaptation to Indigenous contexts.

While international evidence provides a valuable foundation, its most significant influence lies in being translated into locally actionable strategies. Addressing Australia's specific healthcare landscape, workforce distribution and policy context necessitates responsive, multilevel interventions [12]. The international findings within this review emphasise the importance of tailored approaches in Australian rural health policy and practice, while also tackling underrepresentation in the workforce. Consequently, this involves not only workforce planning but also social justice and reconciliation, highlighting the need for inclusive, locally responsive approaches in Australian rural health policy [43, 53].

### 4.7 Methodological Reflection and Limitations

This review's methodology, based on Arksey and O'Malley's scoping review framework and PRISMA‐ScR guidelines, [17, 54] provided a systematic approach to analysing rural nurse retention factors. Including diverse study designs strengthened the researchers' understanding of retention dynamics across different healthcare contexts. However, the heterogeneous nature of the included studies required careful thematic analysis and cross‐validation procedures to ensure a reliable synthesis of findings.

Several key limitations influence the interpretation of this review's results. The exclusion of studies involving multidisciplinary teams and those examining combined regional, rural and remote settings enabled focused analysis but may limit the findings' generalisability. Additionally, restricting the review to English‐language publications potentially overlooks valuable insights from non‐English‐speaking regions facing significant rural workforce challenges. The predominance of studies from high‐income countries may also limit applicability to low‐resource settings.

These methodological constraints suggest clear directions for future research. Subsequent studies should consider broader inclusion criteria encompassing multidisciplinary perspectives and diverse geographical contexts. Expanding language criteria in future reviews would enable a more comprehensive understanding of global rural workforce challenges. Additionally, longitudinal studies examining the long‐term impact of retention interventions would strengthen the evidence base for policy development.

### 4.8 Research Gaps and Future Directions

Strengthening the evidence base for rural nurse retention strategies requires focused research in several priority areas. Longitudinal studies examining rural nurse cohorts over extended periods would enhance understanding of retention initiative effectiveness across career stages. Standardised evaluation frameworks would enable systematic assessment of programme outcomes. These frameworks should incorporate the following:

- Workforce metrics (retention rates, turnover, recruitment success)
- Financial indicators (intervention costs, return on investment)
- Quality measures (job satisfaction, professional development)
- Health service outcomes (care quality, service sustainability)

Economic impact analyses are essential for informed policy development, particularly regarding intervention cost‐effectiveness and broader community benefits. Research must also examine how emerging healthcare delivery models, including digital health initiatives and integrated care approaches, influence rural nursing practice and retention. Understanding these evolving relationships will facilitate the development of evidence‐based retention strategies that support both professional sustainability and community healthcare needs.

Future research efforts should prioritise developing three critical tools:

- a comprehensive evaluation framework for retention strategies,
- implementation guidance for health services addressing organisational context and resource constraints and
- evidence‐based policy recommendations for government agencies.

These practical deliverables will provide actionable pathways for strengthening rural nursing workforce sustainability across micro, meso and macro levels.

## 5 Conclusion

This scoping review enhances understanding of rural nursing workforce sustainability through systematic multilevel analysis. The review's findings demonstrate that effective retention requires coordinated interventions across individual, organisational and systemic levels, driven by evidence‐based strategies and appropriate resource allocation.

The Terry et al. conceptual framework proved valuable for examining the complex interrelationships that influence rural nursing practice. The review's analysis revealed that while personal characteristics and professional identity significantly affect individual decisions, organisational culture and management practices are critical mediators of workforce outcomes. Systemic factors, including educational pathways and government policies, establish the essential context for sustainable workforce development.

Sustainable solutions depend on active partnerships between healthcare organisations, their communities, educational institutions and government agencies. Organisations can strengthen rural healthcare delivery by implementing integrated support systems that combine professional development opportunities with community engagement initiatives.

Evidence from successful programmes demonstrates that effective retention strategies must address both the professional and personal aspects of rural nursing practice. Future research should address identified gaps through longitudinal studies and economic analyses examining intervention effectiveness across rural contexts. These efforts will strengthen the evidence base for developing targeted, cost‐effective approaches to rural nursing workforce sustainability.

## Author Contributions

**Atabong A. Fortabong:** conceptualization (lead), data curation (lead), formal analysis (lead), investigation (lead), methodology (lead), project administration (lead), resources (lead), visualization (lead), writing – original draft (lead). **Natasha Reedy:** supervision (lead), conceptualization (supporting), data curation (supporting), formal analysis (supporting), writing – original draft (supporting), writing – review and editing (supporting). **Blake Peck:** conceptualization (supporting), writing – review and editing (supporting), visualization (supporting). **Kate Kloot:** writing – review and editing (supporting), validation (supporting), visualization (supporting). **Daniel Terry:** supervision (lead), conceptualization (supporting), investigation (supporting), methodology (supporting), validation (supporting), data curation (supporting), formal analysis (supporting), writing – original draft (supporting), visualization (supporting), writing – review and editing (supporting).

## Conflicts of Interest

The authors declare no conflicts of interest.

## Data Availability

The authors have nothing to report.

## Detailed Literature Search Strategy

### APA PsycINFO‐Via EBSCOhost

(TI (Effective* OR Efficac* OR success* OR Benefit* OR strateg* OR intervention*) or AB (Effective* OR Efficac* OR success* OR Benefit* OR strateg* OR intervention*) OR KW (Effective* OR Efficac* OR success* OR Benefit* OR strateg* OR intervention*)) AND (S1 AND S2 AND S3 AND S4).

### CINAHL Ultimate (Via EBSCOhost)

((MH Nurses+) OR ‘nursing workforce’ OR nurs* OR ‘nursing staff*’) AND ((MH ‘Personnel Turnover+’) OR (MH ‘Work Engagement+’) OR (MH ‘Occupational stress+’) OR attrition* OR retain* OR retention* OR recruit* OR turnover* OR ‘intention to leave’) AND ((MH ‘Rural Nursing+’) OR rural* OR remot* OR regional* OR country*) AND ((TI effective* OR AB effective*) OR (TI efficac* OR AB efficac*) OR (TI strateg* OR AB strateg*) OR (TI intervention* OR AB intervention*)).

### Google Scholar (Advanced Search)

With all of the words: Effectiveness OR Efficacy OR Adequacy OR Usefulness OR Successfulness OR Benefit OR Value OR Mitigate OR Retain* OR retention* OR Sustain* OR Recruit* OR Attract* OR Resilien* OR Interven* OR turnover* OR Thrive* OR Leav* ‘Intention to leave’ OR ‘Lived experience’ OR Rural OR Country.

With the exact phrase: ‘nursing workforce’ AND ‘retention strategies’ OR ‘rural health’.

### PubMed

(((‘Nurses’[MeSH Terms] OR ‘nursing workforce’[Text Word] OR ‘nurs*’[Text Word] OR ‘nursing staff*’[Text Word]) AND (‘Personnel Turnover’[MeSH Terms] OR ‘Work Engagement’ [MeSH Terms] OR ‘Occupational stress’[MeSH Terms] OR ‘attrition*’[Text Word] OR ‘retain*’[Text Word] OR ‘retention*’[Text Word] OR ‘recruit*’[Text Word] OR ‘turnover*’[Text Word] OR ‘intention to leave’[Text Word])) AND (‘Rural Nursing’[MeSH Terms] OR ‘rural*’[Text Word] OR ‘remot*’[Text Word] OR ‘regional*’[Text Word] OR ‘country*’[Text Word])) AND (‘effective*’[Text Word] OR ‘efficac*’[Text Word] OR ‘strateg*’[Text Word] OR ‘intervention*’[Text Word]).

### Scopus

(INDEXTERMS (nurses) OR TITLE‐ABS (‘nursing workforce’) OR TITLE‐ABS (nurs*) OR TITLE‐ABS (‘nursing staff*’)) AND (INDEXTERMS (‘personnel turnover’) or INDEXTERMS (‘work Engagement’) OR INDEXTERMS (‘occupational stress’) OR TITLE‐ABS (attrition*) OR title‐abs (retain*) OR TITLE‐ABS (retention*) or TITLE‐ABS (recruit*) OR TITLE‐ABS (turnover*) OR TITLE‐ABS (‘intention to leave’)) AND (INDEXTERMS (‘rural nursing’) OR TITLE‐ABS (rural*) OR title‐abs (remot*) or TITLE‐ABS (regional*) OR TITLE‐ABS (country*)) AND (TITLE‐ABS (effective*) OR TITLE‐ABS (efficac*) OR TITLE‐ABS (strateg*) OR TITLE‐ABS (intervention*)) AND (LIMIT‐TO (SUBJAREA, ‘NURS’)).

### Web of Science/Knowledge

Nurse OR nurse OR ‘nursing profession*’ OR ‘nursing workforce’ (Topic) AND ‘Personnel Turnover’ OR ‘Personnel Shortage’ OR ‘Personnel Retention’ OR ‘Personnel Recruitment’ or Attrition OR Retain* OR Retention* OR Recruit* OR turnover* (Topic) AND ‘Rural Nurses’ OR ‘Rural Nursing’ OR rural* OR remot* OR regional OR Country (Topic) AND Effective* OR Efficac* OR success* OR Benefit* OR strateg* OR intervention* (Topic).
